# Mixed cryoglobulinemia

**DOI:** 10.1186/1750-1172-3-25

**Published:** 2008-09-16

**Authors:** Clodoveo Ferri

**Affiliations:** 1 Dipartimento Medicine e Specialità Mediche, Cattedra ed U.O.C. di Reumatologia, Università di Modena & Reggio Emilia, Modena, Italy

## Abstract

Mixed cryoglobulinemia (MC), type II and type III, refers to the presence of circulating cryoprecipitable immune complexes in the serum and manifests clinically by a classical triad of purpura, weakness and arthralgias. It is considered to be a rare disorder, but its true prevalence remains unknown. The disease is more common in Southern Europe than in Northern Europe or Northern America. The prevalence of 'essential' MC is reported as approximately 1:100,000 (with a female-to-male ratio 3:1), but this term is now used to refer to a minority of MC patients only. MC is characterized by variable organ involvement including skin lesions (orthostatic purpura, ulcers), chronic hepatitis, membranoproliferative glomerulonephritis, peripheral neuropathy, diffuse vasculitis, and, less frequently, interstitial lung involvement and endocrine disorders. Some patients may develop lymphatic and hepatic malignancies, usually as a late complication. MC may be associated with numerous infectious or immunological diseases. When isolated, MC may represent a distinct disease, the so-called 'essential' MC. The etiopathogenesis of MC is not completely understood. Hepatitis C virus (HCV) infection is suggested to play a causative role, with the contribution of genetic and/or environmental factors. Moreover, MC may be associated with other infectious agents or immunological disorders, such as human immunodeficiency virus (HIV) infection or primary Sjögren's syndrome. Diagnosis is based on clinical and laboratory findings. Circulating mixed cryoglobulins, low C4 levels and orthostatic skin purpura are the hallmarks of the disease. Leukocytoclastic vasculitis involving medium- and, more often, small-sized blood vessels is the typical pathological finding, easily detectable by means of skin biopsy of recent vasculitic lesions. Differential diagnoses include a wide range of systemic, infectious and neoplastic disorders, mainly autoimmune hepatitis, Sjögren's syndrome, polyarthritis, and B-cell lymphomas. The first-line treatment of MC should focus on eradication of HCV by combined interferon-ribavirin treatment. Pathogenetic treatments (immunosuppressors, corticosteroids, and/or plasmapheresis) should be tailored to each patient according to the progression and severity of the clinical manifestations. Long-term monitoring is recommended in all MC patients to assure timely diagnosis and treatment of the life-threatening complications. The overall prognosis is poorer in patients with renal disease, liver failure, lymphoproliferative disease and malignancies.

## Disease name and synonyms

Mixed cryoglobulinemia (type II or type III), cryoglobulinemic vasculitis.

## Definition

**Figure 1:**
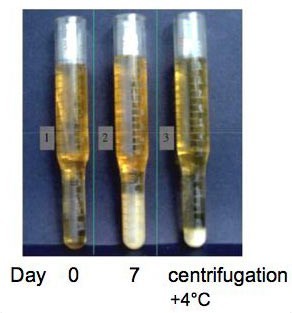
**Cryocrit determination in a patient with mixed cryoglobulinemia (MC)**. Graduated glass tubes with serum sample from cryoglobulinemic patient at different time intervals: 0- soon after serum separation from the whole blood sample (at least 20 ml of whole blood); 7- after 7 days at +4°C; and cryocrit measurement after serum centrifugation, always at +4°C. (modified from [24], Ferri C *et al*, Sem Arthritis Rheum 2004, 33:355–74.).

The term cryoglobulinemia refers to the presence in the serum of one (monoclonal cryoimmunoglobulinemia) or more immunoglobulins (mixed cryoglobulinemia), which precipitate at temperatures below 37°C and re-dissolve on re-warming [1,2]. This is an *in vitro *phenomenon (Fig. 1), the actual mechanism(s) of cryoprecipitation remains obscure, it could be secondary to intrinsic characteristics of both mono- and polyclonal immunoglobulin (Ig) components, it can be caused as well by the interaction among single components of the cryoprecipitate [1-14].

Cryoglobulinemia is usually classified into three subgroups [4] according to Ig composition (Table 1): type I cryoglobulinemia is composed of only one isotype or subclass of immunoglobulin. Both type II and type III mixed cryoglobulins are immune complexes composed of polyclonal IgGs, the autoantigens, and mono- or polyclonal IgMs, respectively; the IgMs are the corresponding autoantibodies with rheumatoid factor (RF) activity [3-6]. With more sensitive methodologies, *i.e*. immunoblotting or 2-dimensional polyacrylamide gel electrophoresis, type II mixed cryoglobulins frequently shows a microheterogeneous composition; in particular, oligoclonal IgM or a mixture of polyclonal and monoclonal IgM can be detected [3]. This particular serological subset, termed type II-III mixed cryoglobulinemia (MC), could represent an intermediate, evolutive state from type III to type II MC. Moreover, type II-III MC could fit together the most recent molecular studies showing the presence of oligoclonal B-lymphocyte proliferation in the liver and bone marrow biopsies from MC patients [3]. In two third of type II MC, a cross-idiotype WA monoclonal RF (firstly isolated from the serum of a patient with Waldenström's macroglobulinemia) has been demonstrated [14].

**Table 1:** Classification and clinico-pathological characteristics of different cryoglobulinemias.

	**Composition**	**Pathological findings**	**Clinical associations**
**Type I cryoglobulinemia**	monoclonal Ig, mainly IgG, or IgM, or IgA self-aggregation through Fc fragment of Ig	tissue histological alterations of underlying disorder	-lymphoproliferative disorders: MM, WM, CLL, B-cell NHL
**Type II** **mixed cryoglobulinemia**	monoclonal IgM (or IgG, or IgA) with RF activity (often cross-idiotype WA-mRF) and polyclonal Ig (mainly IgG)	-leukocytoclastic vasculitis -B-lymphocyte expansion with tissue infiltrates	-infections (mainly HCV) -autoimmune/lymphoproliferative disorders -rarely 'essential'
**Type II–III** **mixed cryoglobulinemia**	oligoclonal IgM RF or mixture of poly/monoclonal IgM (often cross-idiotype WA-mRF)	-leukocytoclastic vasculitis -B-lymphocyte expansion with tissue infiltrates	-infections (mainly HCV) -autoimmune/lymphoproliferative disorders -rarely 'essential'
**Type III** **mixed cryoglobulinemia**	polyclonal mixed Ig (all isotypes) with RF activity of one polyclonal component (usually IgM)	-leukocytoclastic vasculitis -B-lymphocyte expansion with tissue infiltrates	-infections (mainly HCV) -more often autoimmune disorders -rarely 'essential'

lymphoproliferative disorders: MM (multiple myeloma), WM (Waldenstrom's macroglobulinemia), chronic lymphocytic leukemia), B-cell non-Hodgkin's lymphoma;

Ig: immunoglobulin; RF: rheumatoid factor; HCV: hepatitis C virus

**Figure 2:**
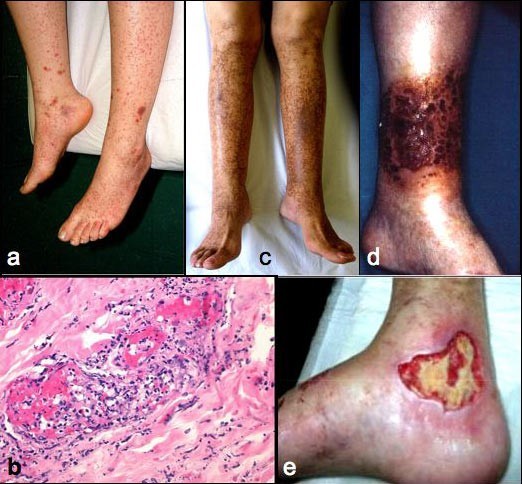
**Cutaneous manifestations of mixed cryoglobulinemia (MC)**. (a) recent onset orthostatic purpura; at this stage the histopathological evaluation shows (b) the classical necrotizing leukocytoclastic vasculitis characterized by diffuse fibrinoid necrosis and disintegrated neutrophil permeation of the vessel walls; (c) symmetrical hyperpigmentation of the skin on the legs after repeated episodes of purpura; both orthostatic purpura and these permanent ochraceous lesions represent the typical skin manifestations of MC; (d) severe vasculitic manifestation; (e) wide skin ulcer, often resistant to treatment.

Type I cryoglobulinemia is almost invariably associated with well-known hematological disorders and is frequently asymptomatic *per se*; similarly, circulating mixed cryoglobulins are commonly detected in a great number of infectious or systemic disorders [1-14]. On the contrary, 'essential' MC represents a distinct disorder [3-6], which can be classified among systemic vasculitides [3-10]. Cryoglobulinemic vasculitis (Fig. 2) is secondary to vascular deposition of circulating immune-complexes, mainly cryoglobulins, and complement, with the possible contribution of both hemorheological and local factors [3-6]. Due to its clinical and histological features, MC is classified in the subgroup of small vessel systemic vasculitides, which also includes cutaneous leukocytoclastic vasculitis and Henoch-Schonlein purpura [3,10].

The leukocytoclastic vasculitis is the histopathological hallmark of MC (Fig. 2). It may involve small- and medium-sized vessels and may be responsible for multiple organ involvement. The term 'cryoglobulinemic vasculitis' is frequently used as synonym; it better focus on the typical histopathological alterations responsible for cutaneous and visceral organ involvement [3,10].

## Epidemiology

**Figure 3:**
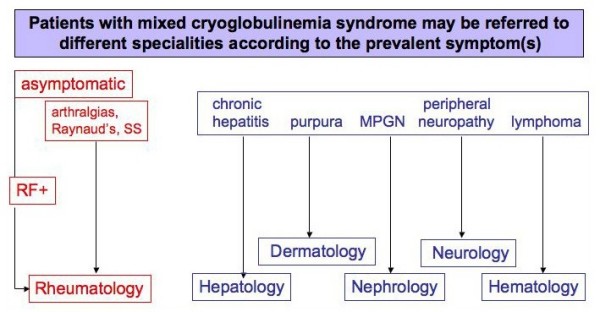
**Different referrals of patients with mixed cryoglobulinemia (MC)**. Given its clinical polymorphism, the MC syndrome may develop through different, often unpredictable symptoms. Consequently, MC patients may be referred to different specialties according to prevalent or apparently unique clinical manifestation, such as membranoproliferative glomerulonephritis (MPGN) or purpuric skin lesions. Patients with very mild manifestations, often arthralgias and/or serum rheumatoid factor (RF) positivity, are generally referred to rheumatologic clinic.

The prevalence of MC presents great geographic heterogeneity; the disease is more common in Southern Europe than in Northern Europe or Northern America [3-13]. It is considered to be a rare disorder, however, yet there are no adequate epidemiological studies regarding its overall prevalence. Given its clinical polymorphism, a single manifestation (skin vasculitis, hepatitis, nephritis, peripheral neuropathy, *etc*.) is often the only apparent or clinically predominant feature, so that MC patients are often referred to different specialties (Fig. 3). A correct diagnosis might thus be delayed or overlooked entirely. Consequently, the actual prevalence of MC might be underestimated.

For the same reasons, the clinical pattern of the MC syndrome may vary largely among patients series referred to different tertiary care facilities [3,6,8-14] (Fig. 3).

It has been estimated that low levels of circulating mixed cryoglobulins can be detected in over 50% of HCV-infected individuals, while overt cryoglobulinemic syndrome develops in about 5% [3,15,16]. Because of the wide diffusion of HCV infection worldwide, a growing incidence of HCV-related MC can be expected, especially in underdeveloped countries where HCV in the general population is rather prevalent [3,17].

The prevalence of 'essential' MC is reported as approximately 1:100,000 but this term is now referred to a minority of MC patients. The female-to-male ratio is 3:1.

## Clinical description

According to its first description [5], MC syndrome is characterized clinically by a triad -purpura, weakness, arthralgias- and by a series of pathological conditions, including chronic hepatitis, membranoproliferative glomerulonephritis (MPGN), peripheral neuropathy, skin ulcers, diffuse vasculitis, and, less frequently, by lymphatic and hepatic malignancies [3-12,17-23]. The clinical pattern of cryoglobulinemic vasculitis is comparable in patients with type II or type III MC [3]. The prevalence of MC manifestations reported in Table 2 regards an Italian patient population referred to our university-based division of rheumatology. Patients' recruitment at different specialist centers, as well as the racial composition among patients' series may be responsible for the variable prevalence of MC symptoms reported in the literature [3-10,14].

**Table 2:** Demographic, clinico-serological and virological features of 250 mixed cryoglobulinemia (MC) patients*.

**Clinical features**	
Age at disease onset, mean ± SD yrs (range)	54 ± 13 (29–72)
Female/Male ratio	3
Disease duration, mean ± SD years (range)	12 ± 10 (1–40)
Purpura	98%
Weakness	98%
Arthralgias	91%
Arthritis (non-erosive)	8%
Raynaud's phenomenon	32%
Sicca syndrome	51%
Peripheral neuropathy	81%
Renal involvement**	31%
Liver involvement	73%
B-cell non-Hodgkin's lymphoma	11%
Hepatocellular carcinoma	3%
Cryocrit, mean ± SD %	4.4 ± 12
Type II/type III mixed cryoglobulins	2/1
C3, mean ± SD mg/dl (normal 60–130)	93 ± 30
C4, mean ± SD mg/dl (normal 20–55)	10 ± 12
Antinuclear antibodies	30%
Antimitochondrial antibodies	9%
Anti-smooth muscle antibodies	18%
Anti-extractable nuclear antigen antibodies	8%
anti-HCV Ab ± HCV RNA, %	92%
Anti-HBV antibodies	32%
HBsAg	1%

* evaluated at the end of the patients' follow-up

**invariably membranoproliferative glomerulonephritis

**Figure 4:**
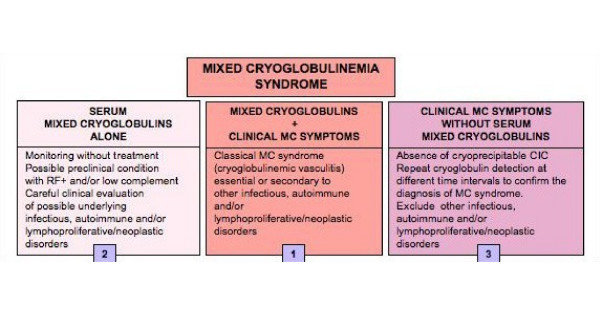
**Relationship between cryoglobulin detection and overt mixed cryoglobulinemia (MC) syndrome**. (1) Definite MC syndrome (or cryoglobulinemic vasculitis) is a combination of serological findings (mixed cryoglobulins with RF activity and frequent low C4) and typical clinico-pathological features (purpura, leukocytoclastic vasculitis, and frequent multiple organ involvement); see also Table 3. However, incomplete MC syndrome can be observed at any time during the natural history of the disease; (2) Isolated serological alterations may be detected in the early stages of the disease or during the clinical remission; on the contrary; (3) the absence of serum cryoglobulins in patients with overt MC syndrome may be a transient phenomenon due to the wide variability of the percentage of cryoprecipitable immune-complex during the natural history of the disease or, less frequently, to a switching from 'benign' B-cell lymphoproliferation to malignant lymphoma.

The presenting symptoms of MC largely vary among patients with cryoglobulinemia. At the initial observation, MC shows different clinico-serological patterns, varying from apparently isolated serum mixed cryoglobulins to complete cryoglobulinemic syndrome (Fig. 4). This is a combination of serological findings (mixed cryoglobulins with RF activity and frequent low C4) and clinico-pathological features (purpura, leukocytoclastic vasculitis with multiple organ involvement) [6-10,14,24,25]. Asymptomatic serum mixed cryoglobulins can be found in some chronically HCV-infected individuals [3,15,16]; a condition that may precede for years or decades the clinical onset of the disease. On the other hand, some patients show typical cryoglobulinemic syndrome, without serum cryoglobulins, the hallmark of the disease (Fig. 4). This is generally a transient phenomenon due to the wide variability of the percentage of cryoprecipitable immune-complex [3,26]. Repeated cryoglobulin determinations are necessary for a correct diagnosis in these subjects.

**Skin lesions **represent the most frequent manifestations of the MC [6-10,14,24,25]. Orthostatic purpura is generally intermittent, the dimension and diffusion of purpuric lesions largely varied, from sporadic isolated petechias to severe vasculitic lesions, often complicated by torpid ulcers of the legs and malleolar areas (Fig. 2). In a significant proportion of patients, repeated episodes of purpura may lead to stable, often confluent areas of ochreous coloration on the legs (Fig. 2). Cutaneous manifestations, in particular orthostatic purpura and ulcers, are the direct consequence of vasculitic alterations with the possible contributions of various co-factors, in particular chronic venous insufficiency, physical stress, mainly the prolonged standing, and/or muggy weather. In addition, the contribution of hemorheological disturbances due to high cryocrit levels may also be taken in account [27]. In this respect, the purpuric outbreaks are frequently observed late in the afternoon when highest cryocrit levels are generally observed, often in concurrence with prolonged standing [24].

MC patients commonly report **arthralgias**, while clear clinical signs of arthritis (usually mild, non-erosive oligoarthritis) are relatively rare [11,12,25,28]

Almost half MC patients complain of **xerostomia and xerophthalmia**, however, only a few cases meet the current criteria for the classification of primary Sjogren's syndrome (see 'differential diagnosis') [11,12,25].

**Peripheral neuropathy **may frequently complicate the clinical course of the MC, in the majority of cases as mild sensory neuritis [24,25,29,30]. The common symptoms are paresthesias with painful and/or burning sensations in the lower limbs, often with nocturnal exacerbation. The patient quality of life may be severely compromised because of the chronicity of these symptoms along with their scarce sensibility to therapeutic attempts. In a minority of cases the peripheral neuropathy may be complicated by severe sensory-motor manifestations, which usually appeared abruptly, often as asymmetric mononeuritis; in some patients, it may complicate the alpha-interferon treatment, possibly in predisposed subjects [29-33]. Dysarthria and hemiplegia expression of central nervous system involvement are rarely reported [25,34]; it is often difficult to distinguish these symptoms from the most common atherosclerotic manifestations.

Since HCV infection represents the underlying disorder of the MC in the vast majority of cases, **overt chronic hepatitis**, generally with mild-to-moderate clinical course, can be observed at any time during the natural history of the disease [24,25]. Chronic hepatitis was found in a great number of patients (Table 2), evolving to cirrhosis in 1/4, while only 7 patients developed hepatocellular carcinoma. In few cases, especially in combination with renal involvement, liver involvement became a life-threatening complication. On the whole, the clinical course and the prognostic value of this manifestation seem to be less severe if compared to HCV-related chronic hepatitis without MC syndrome [24,25]; similarly, hepatocellular carcinoma less frequently complicates MC syndrome compared to the whole population of HCV-positive individuals [24,25]. These differences are quite intriguing, but very difficult to fully explain. It is possible that the light consumption of alcohol and/or the relatively low prevalence of HCV genotype 1b [24,25] may explain, at least in part, the rather benign clinical course of liver involvement and its scarce prognostic relevance in MC series.

**Membranoproliferative glomerulonephritis type 1 **is another important organ involvement, which may severely affect the prognosis and survival of the disease [24,25,34-37]. MC-related nephropathy is a typical immune-complex-mediated glomerulonephritis, although other immunological mechanisms have also been hypothesized [24,25,38,39].

**Widespread vasculitis **involving medium-small sized arteries, capillaries and venules with multiple organ involvement may develop in a small proportion of patients [3,6,10,24,25,40]. This extremely severe complication may involve the skin, kidney, lungs, central nervous system, and gastrointestinal tract. In rare cases intestinal vasculitis may suddenly complicate the disease, often in patients with renal and/or liver involvement; pain simulating an acute abdomen is the presenting symptom of intestinal vasculitis. A timely diagnosis and aggressive steroid treatment are necessary for this life-threatening complication.

**Interstitial lung involvement **has been anecdotally observed in MC syndrome as well as in patients with isolated HCV infection [3,25,41-43]. Almost invariably, lung involvement in MC is characterized by subclinical alveolitis, as demonstrated by means of bronco-alveolar lavage in unselected patient series [44]; this condition may predispose to harmful infectious complications and, in rare cases, it may lead to clinically evident interstitial lung fibrosis. The hyperviscosity syndrome, due to high levels of serum cryoglobulins is another rare clinical manifestation of MC [27]. Generally, there was no relationship between the severity of clinical symptoms, such as glomerulonephritis, skin ulcers, or diffuse vasculitis and the serum levels of cryoglobulins and/or hemolytic complement [3,24,25]. Low complement activity is almost invariably detectable in MC, with the typical pattern of low or undetectable C4 and normal or relatively normal C3 serum levels, regardless the disease activity (Table 2). Moreover, *in vitro *consumption of complement can be also observed due to the anti-complement activity of some cryoimmunoglobulins [3]. Of interest, a sudden increase in C4, raised to abnormally high levels, can be observed in MC patients developing a B-cell lymphoma [45]. The lack of correlation between circulating cryoglobulin levels and the severity/activity of MC manifestations might be explained on the basis of different hypotheses: the pathogenic role of other non-cryoprecipitable immune-complexes, their intrinsic capacity to activate the complement, and/or the *in situ *formation of immune-complexes, with a relative concentration of HCV virions [3,14,24,25].

Some **endocrinological disorders **are significantly more frequent in MC patients compared to the general population [3,12,13,24,25,46-50]. The most common thyroid disorders are autoimmune thyroiditis, subclinical hypothyroidism, and thyroid cancer; while hyperthyroidism is less frequent, generally as reversible complication of interferon treatment [51-54]. Moreover, a statistically increased incidence of diabetes mellitus has been observed in HCV-positive patients with and without MC syndrome compared to the general population [55-57]. Finally, HCV-positive males with or without cryoglobulinemic vasculitis may develop erectile dysfunction, attributable to hormonal and/or neuro-vascular alterations [49].

**B-cell lymphom**a is the most frequent neoplastic manifestation complicating MC, often as late manifestation of the MC syndrome [58-65]. This complication may be related to peripheral B-lymphocyte expansion and to lymphoid infiltrates observed in the liver and bone marrow of MC patients [19,39,58,59]. In particular, these infiltrates have been regarded by some authors as "early lymphomas", since they are sustained by lymphoid components indistinguishable from those of B-cell chronic lymphocytic leukemia/small lymphocytic lymphoma (B-CLL) and immunocytoma (Ic) [3,12,25,59]. However, unlike frank malignant lymphomas, they tend to remain unmodified for years or even decades and are followed by overt lymphoid tumors in about 10% of cases [3]. These characteristics justify the proposed term of "monotypic lymphoproliferative disorder of undetermined significance (MLDUS)" [3,12,25,59]. Interestingly enough, type II MC-related MLDUS has its highest incidence in the same geographic areas where about 30% of patients with 'idiopathic' B-cell lymphomas also display HCV-positivity, and where an increased prevalence of HCV genotype 2a/c has been observed in both MC and lymphomas [3,12,25,59]. The type II MC-associated MLDUS presents two main pathological patterns; namely, the B-CLL-like and the Ic-like [3,12,25,59]. In the clinical practice, it is not rare to observe the appearance of malignant B-cell lymphomas in patients with mild MC clinical course, sometimes unexpectedly during a routine evaluation. It is possible to observe a sudden decrease or disappearance of serum cryoglobulins and RF, sometimes associated with abnormally high levels of C4, as presenting symptom of complicating B-cell malignancy [40].

Other neoplastic manifestations, *i.e*. hepatocellular carcinoma or papillary thyroid cancer, are less frequently observed [3,11,24,50,59]. In this light, the MC can be regarded as a pre-neoplastic disorder [61]; consequently, a careful clinical monitoring is recommendable, even in the presence of mild MC syndrome [3,11].

## Etiopathogenesis

**Figure 5:**
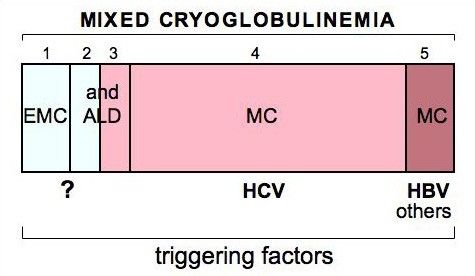
**Schematic representation of different clinical and virological subsets of mixed cryoglobulinemia (MC) syndrome**. 1) 'essential' MC (EMC); 2) and 3) 'essential' MC and HCV-associated MC syndrome in the setting of definite autoimmune-lymphoproliferative disorders (ALD), such as autoimmune hepatitis, Sjögren's syndrome or B-cell lymphomas; 4) the most frequent subset of HCV-associated MC syndrome; 5) MC associated with other infectious agents such as hepatitis B virus (HBV). (modified from [12,13]: Ferri C *et al*, B-cells and mixed cryoglobulinemia. *Autoimm Rev *2007, 7: 114–20; Mascia MT *et al*, Non HCV-related mixed cryoglobulinemia. *Dig Liver Dis *2007, 39: S61–4.).

Since the first description of MC syndrome [5], chronic hepatitis has been reported as frequent manifestation appearing during the clinical course of the disease [3-6,11,24]; therefore, a possible role for hepatotropic viruses in the pathogenesis of the disease has long been suggested [3,12]. A role for hepatitis B virus (HBV) has been firstly investigated [66]; however, HBV viremia is rarely recorded, while anti-HBV antibodies largely varied among different MC patient populations [3]. It can be estimated that HBV can represent a causative factor of MC in less than 5% of individuals (Fig. 5).

Soon after the discovery of hepatitis C virus (HCV) as the major etiologic agent of non-A-non-B chronic hepatitis [67], a possible role of HCV infection in MC was proposed independently by two pioneering reports [68,69] showing a significantly higher prevalence of serum anti-HCV antibodies compared to the general population. This hypothesis was definitely demonstrated in 1991, when the presence of HCV RNA was detected by means of polymerase chain reaction (PCR) in 86% of Italian MC patients [70]. Successively, an increasingly number of studies including clinico-epidemiological observations, as well as both histopathological and virological investigations (HCV RNA detection by PCR and/or *in situ *hybridization) have been definitely established the preeminent role of HCV in the pathogenesis of MC [8-14,21,25,59,71-76]. The prevalence of serum anti-HCV antibodies and/or HCV RNA in MC patients ranged from 70% to almost 100% among different patient populations [3,11-13,25]. Given the striking association between MC and HCV infection, the term 'essential' is now referred to a minority of MC patients (in Italy <5%, Fig. 5) [3,11-13,25].

**Figure 6:**
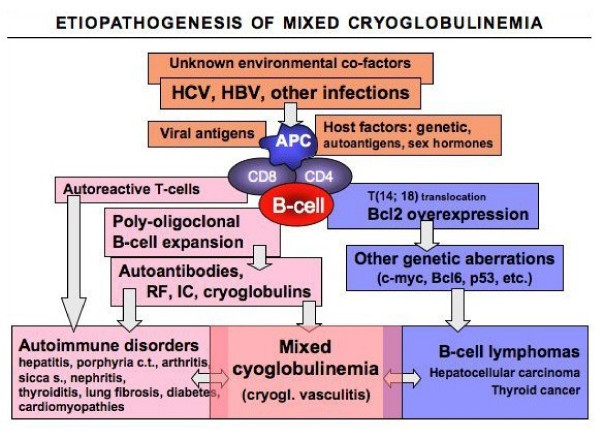
**Etiopathogenesis of mixed cryoglobulinemia (MC) syndrome**. The figure summarizes the etiopathogenetic cascade of MC and other HCV-related disorders. This is probably a multifactorial and multistep process: the remote events include some infectious agents, mainly HCV, predisposing host factors, and possible unknown environmental/toxic triggers. Viral antigens (for example HCV core, envelop E2, NS3, NS4, NS5A proteins) may exert a chronic stimulus on the host immune- system through specific lymphocyte receptors, such as CD81 that may interact with the viral E2. Predisposing host factors may include particular HLA alleles, metabolic and hormonal conditions. The main consequence is a 'benign' B-cell proliferation with a variety of autoantibody production, among which rheumatoid factor (RF), and cryo- and non-cryoprecipitable immune-complexes (IC). These serological alterations may be correlated with different organ- and non-organ-specific autoimmune disorders, including the MC syndrome (or cryoglobulinemic vasculitis). Moreover, the activation of Bcl2 proto-oncogene, responsible for prolonged B-cell survival, may be a predisposing condition to other genetic aberrations, which may lead to frank B-cell lymphomas and other malignancies. The appearance of malignant neoplasias can be observed in a small but significant percentage of patients, usually as late complication. Both immunological and neoplastic disorders show a clinico-serological and pathological overlap. Often, autoimmune organ-specific manifestations may evolve to systemic conditions, and less frequently to malignancies. Conversely, it is not rare that patients with malignancies may develop one or more autoimmune manifestations. In this scenario, MC syndrome represents a crossing road between autoimmune and neoplastic disorders. (modified from [11,12]: Ferri C *et al*, HCV-related autoimmune and neoplastic disorders: the HCV syndrome. *Dig Liver Dis *2007, 39: S13–21; Ferri C *et al*, B-cells and mixed cryoglobulinemia. *Autoimm Rev *2007, 7: 114–20.).

Because of the frequent association between MC and HCV, the behavior of MC is closely linked to the natural history of HCV chronic infection [3,11-13,24,25]. However, MC is also the result of concomitant genetic and/or environmental factors, which remain largely unknown (Fig. 6). HCV has been recognized to be both a hepato- and lymphotropic virus, as suggested by the presence of active or latent viral replication in the peripheral lymphocytes of patients with type C hepatitis or MC [3,73,77]. HCV is an RNA virus without reverse trascriptase activity; therefore viral genome cannot integrate in the host genome [63]. Probably, HCV may exert a chronic stimulus of the immune-system, through different viral proteins such as core protein [9,22,63]. Chronic stimulation of the lymphatic system exerted through viral epitopes, autoantigen production, and/or molecular mimicry mechanism has been suggested by the presence, in HCV-positive patients, of anti-GOR antibodies, which are cross-reactive autoantibodies directed to both HCV core and a nuclear antigen named GOR [3,59,63]. Other Authors suggested that HCV, in association with very low-density lipoprotein (VLDL), might induce a T-independent primordial B-cell population producing monoclonal immunoglobulin with WA idiotype [14]. In turn, HCV-VLDL complexes may trigger RF production as consequence of somatic mutations of WA clones; the possible evolution to B-cell lymphomas might be the consequence of the accumulation of stochastic genetic aberrations [59]. Chronic stimulation of the B-cell by HCV epitopes may produce the expansion of some B-cell subpopulations with favorable and/or dominant genetic characteristics. This hypothesis recalls the pathogenetic role of *Helicobacter pylori *in MALT lymphoma of the stomach, for which different evolutive phases are requested [59].

Another important pathogenetic factor is the interaction between HCV E2 protein and CD81 molecule, a quite ubiquitary tetraspannin present on B-cells surface [78]; the consequence may be the strong and sustained polyclonal stimulation of B-cell compartment (Fig. 6). A following pathogenetic step of HCV-related autoimmune-lymphoproliferative disorders may be the t [14,18] translocation observed in B-cells of HCV-infected individuals [63,79]. Even if not definitely confirmed [80,81], the t [14,18] translocation might lead to abnormally elevated expression of Bcl-2 protein with consequent inhibition of apoptosis and abnormal B-cell survival. Interestingly, the relevant prevalence of t [14,18] translocation in patients with only type C hepatitis (about 37–38%), become particularly high in patients with HCV-related cryoglobulinemic syndrome, ranging 85% in type II MC [63]. It is possible to hypothesize that during chronic HCV infection, several factors (including the interaction between HCV E2 protein and CD81 molecule, the high viral variability, and the persistent infection of both hepatic and lymphatic cells) may favor a sustained and strong B-cell activation (Fig. 6). This latter may in turn favors the apparition of t [14,18] translocation and Bcl-2 overexpression; the consequent B-lymphocyte expansion is responsible for autoantibody production, including the cryoglobulins [3,11,12,63,77-79]. In addition, the prolonged B cell survival may represent a predisposing condition for further genetic aberrations, which may lead to frank B-cell malignancy as late complication of MC syndrome [11,20,24,82].

Of interest, HCV-driven lymphoproliferation may explain the pathogenetic role of HCV infection also in 'idiopathic' B-cell lymphomas [11,59,64,65,80]. This association was firstly described in unselected Italian patients with 'idiopathic' B-cell lymphomas [64] and successively confirmed by different epidemiological and laboratory studies, mainly in the same geographical areas where HCV-associated MC is commonly found [65].

Given its biological characteristics, HCV may be involved in a wide number of autoimmune and lymphoproliferative disorders [3,11,12,63,77-79]. Fig. 6 summarizes the main causative factors -infectious, toxic, genetic, and/or environmental- that are potentially involved in the pathogenesis of MC [11,12,79,82-84]. These factors, alone or in combination, may trigger two multistep pathogenetic processes, not mutually exclusive, responsible for MC and other HCV-related disorders. The first one produces a 'benign' poly-olygoclonal B-lymphocyte proliferation responsible for organ- and non-organ-specific autoimmune disorders, including the immune-complex-mediated cryoglobulinemic vasculitis; the second one is characterized by different oncogenetic alterations, which ultimately may lead to malignant complications [11,82]. Comparable pathogenetic mechanisms could be also hypothesized for HCV-negative MC, this intriguing clinical subset might be correlated to other infectious agents or associated to some well-known autoimmune/rheumatic or lymphoproliferetive disorders (Fig. 5, 6) [13]. Th1-mediated tissue alterations are probably responsible for some endocrine disorders, i.e. diabetes type 2 and thyroid dysfunction, in HCV-associated MC patients [57]. Our preliminary data suggest that HCV infection of thyrocytes, as well as of beta cells, may upregulate CXCL10 gene expression and secretion (as shown in human hepatocytes); the consequent recruitment of Th1 lymphocytes that secrete IFN-γ and TNF-α may in turn induce CXCL10 secretion by infected cells, thus perpetuating the immune cascade responsible for these endocrine disorders. All the above pathogenetic mechanisms could be also hypothesized for HCV-negative MC, this intriguing clinical subset might be correlated to other infectious agents or associated to some well-known autoimmune/rheumatic or lymphoproliferetive disorders (Fig. 5, 6) [13].

MC with or without overt clinical syndrome has been reported in patients with a great number of infectious agents, usually as anecdotally observations [9]. A significant prevalence of MC has been observed in patients with human immunodeficiency virus (HIV) infection, with and without HCV co-infection [85]. HIV alone may exert a continuous antigenic stimulation of B-lymphocytes; these latter may be responsible for type III MC production earlier in the course of HIV infection. In some patients the B-cell disorder may evolve to monoclonal MC with typical clinical syndrome. As observed for HCV infection, the prevalence of other virus-related MC seems to show a variable prevalence among patient's series from different geographical areas [86]. Moreover, a number of clinico-epidemiological studies revealed a heterogeneous distribution of different HCV-related extra hepatic manifestations, including some autoimmune disorders such as 'primary' Sjogren's syndrome [87-98].

**Figure 7:**
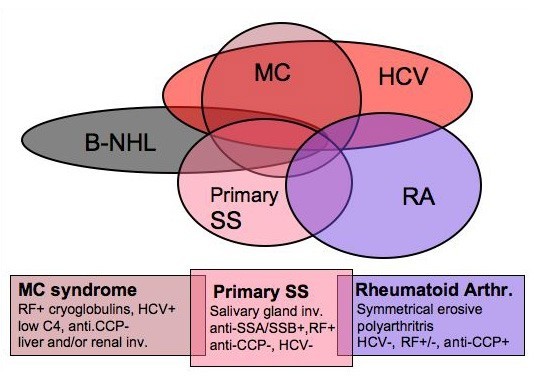
**Differential diagnosis between mixed cryoglobulinemia and other autoimmune-lymphoproliferative disorders in the setting of HCV infection**. Mixed cryoglobulinemia (MC) syndrome, primary Sjögren's syndrome (pSS), and rheumatoid arthritis (RA) show a clinico-pathological overlap, including the possible association with HCV infection. The following parameters may be usefully employed for a correct differential classification/diagnosis: pSS shows typical histopathological pattern of salivary gland involvement and specific autoantibodies (anti-RoSSA/LaSSB), which are rarely found in MC patients; conversely, cutaneous leukocytoclastic vasculitis, visceral organ involvement (glomerulonephritis, hepatitis), low C4, and HCV infection, are typically found in MC. Moreover, erosive symmetrical polyarthritis and serum anti-cyclic citrullinated peptide antibodies (anti-CCP) characterize classical RA. Finally, B-cell non-Hodgkin's lymphoma (B-NHL) may complicate these diseases, more frequently MC and SS. The appearance of B-NHL can be timely suspected by careful clinico-serological monitoring and diagnosed by bone marrow/lymph node biopsies and total body CT scan. RF: rheumatoid factor. (modified from [25]. Ferri C and Mascia MT, *Curr Opin Rheumatol *2006, 18: 54–63, with permission from Lippincott Williams & Wilkins).

Cryoglobulinemic syndrome may share a number of etiopathogenetic events and clinical features with both autoimmune diseases such as autoimmune hepatitis, Sjögren's syndrome, and polyarthritis, and B-cell lymphomas [3,11,12,25,28,85-100]. Therefore, a differential diagnosis should be carefully done in all patients with MC syndrome (Fig. 7, see also the paragraph 'differential diagnosis'); a correct disease classification may decisively affect the overall clinico-therapeutic approach and prognosis.

## Diagnostic methods

There are no available diagnostic criteria for MC; in 1989 the Italian Group for the Study of Cryoglobulinemias has proposed preliminary criteria for MC classification [101]. A revised version of these criteria (Table 3), including clinico-pathological and virological findings, has been successively proposed [3]. Circulating mixed cryoglobulins, low C4, and orthostatic skin purpura are the hallmarks of the disease; moreover, leukocytoclastic vasculitis, involving medium- and, more often, small-sized blood vessels (arterioles, capillaries, and venules) is the typical pathological finding of involved tissues. It is easily detectable by means of skin biopsy of recent vasculitic lesions (within the first 24–48 hours) [3,11,12,25].

**Table 3:** Proposed criteria for the classification of mixed cryoglobulinemia (MC) patients.

Criteria	Serological	Pathological	Clinical
***major***	mixed cryoglobulins low C4	leukocytoclastic vasculitis	purpura
***minor***	rheumatoid factor + HCV + HBV +	clonal B-cell infiltrates (liver and/or bone marrow)	chronic hepatitis MPGN peripheral neuropathy skin ulcers
**definite mixed cryoglobulinemia syndrome:**			
a) serum mixed cryoglobulins (± low C4) + purpura + leukocytoclastic vasculitis			
b) serum mixed cryoglobulins (± low C4)+ 2 minor clinical symptoms + 2 minor serological/pathological findings			
**essential or secondary mixed cryoglobulinemia:**			
absence or presence of well-known disorders (infectious, immunological or neoplastic)			

HCV+ or HBV+: markers of hepatitis C virus or hepatitis B virus infection (anti-HCV ± HCV RNA; HBV DNA or HBsAg); MPGN: membranoproliferative glomerulonephritis.

The detection of serum mixed (IgG-IgM) cryoglobulins is necessary for a correct classification of MC syndrome (Table 3, Fig. 1). Unfortunately, there are no universally accepted methodologies for cryoglobulin measurements, but simple standardized indications are often sufficient for testing cryoglobulinemia [3,9,23]. Since cryoglobulins present a high thermal instability, the measurement of Ig cryoprecipitate should be performed immediately in the same place where the blood is sampled. For a correct evaluation of serum cryoglobulins, it is necessary to avoid false-negative results due to Ig cold precipitation also at room temperature. For this purpose, the first steps (blood sampling, clotting, and serum separation by centrifugation) should be always carried out at 37°C and the cryocrit determination and cryoglobulin characterization at 4°C (after 7 days). The serum with cryoglobulins should be tested for reversibility of the cryoprecipitate by rewarming an aliquot at 37°C for 24 hours. Moreover, cryocrit determinations (percentage of packed cryoglobulins referred to total serum after centrifugation at +4°C; Fig. 1) should be done on blood samples without anticoagulation to avoid false-positive results due to cryofibrinogen or heparin-precipitable proteins. Without the above relatively simple precautions, not only will the quantities of cryoglobulins measured be incorrect, but the test may completely fail to even detect cryoglobulins.

After isolating and washing the cryoprecipitate, the identity of cryoglobulin components can be determined by immunoelectrophoresis or immunofixation. These analysis must be performed at 37°C to avoid precipitation and hence loss of the cryoglobulin during the procedures. More sophisticated methodologies, such immunoblotting or two-dimensional polyacrylamide gel electrophoresis, can be proposed for laboratory investigations [102-104]. While the detection of serum cryoglobulins is fundamental for the diagnosis of MC, the levels of serum cryoglobulins usually do not correlate with the severity and prognosis of the disease [3,11,12,24,25]. Very low levels of cryocrit, often difficult to quantify, can be associated with severe and/or active cryoglobulinemic syndrome; on the contrary, high cryocrit values may characterize a mild or asymptomatic disease course. In rare cases, very high cryocrit levels, possibly associated to cryogel phenomenon, can be associated to classical hyperviscosity syndrome [24,25,27]. On the contrary, a sudden decrease or disappearance of serum mixed cryoglobulins, with or without abnormally high levels of C4, can be regarded as alarming signal of complicating B-cell malignancy [45].

Table 4 summarizes the clinico-serological work-up at patient's first evaluation in order to correctly classify the MC syndrome and to identify possible overlapping disorders (see next paragraph), and/or comorbidities. The prevalence of these latter, in particular atherosclerosis, may be correlated with the disease duration, as well as with cumulative side effects of prolonged treatments. Moreover, the diagnosis and monitoring of the major MC manifestations is fundamental for timely treatments, especially for life-threatening liver, renal, and/or neoplastic complications [3,11,12,24,25].

**Table 4:** Clinico-diagnostic assessment of mixed cryoglobulinemia (MC) syndrome

**Clinical and serological work-up at patient's first evaluation**
• past clinical history, physical examination
• chest x-ray, EKG, abdominal US, blood chemistry & urinalysis
• cryoglobulin detection and characterization (see Tab. 1)
• RF, C3–C4, ANA, anti-ENA, ANCA, ASMA, AMA, anti-LKM1, others auto-Ab
• virological markers: HCV (genotyping), HBV, others
• evaluate possible comorbidities (cardiovascular, endocrine/metabolic, etc.)
• MC classification (definite, essential, secondary): see Tab. 3

**Diagnosis & monitoring of major MC complications**

• chronic hepatitis, cirrhosis, hepatocellular carcinoma: monitoring (every 6–12 month) of ALT, AP & liver US (biopsy, CT scan)
• glomerulonephritis: monitoring of urinalysis & serum creatinine (kidney US, biopsy)
• peripheral neuropathy: clinical monitoring; EMG
• skin ulcers: exclusion of vascular comorbidities (A-V Doppler evaluation)
• sicca syndrome: differential diagnosis with primary SS (see Fig. 7)
• arthritis: differential diagnosis with RA (see Fig. 7)
• thyroid involvement: hormones, auto-Ab, neck US, fine-needle aspiration
• B-cell lymphoma: clinical monitoring; bone marrow/lymph node biopsies, total body CT scan

**Abbreviations: **F: rheumatoid factor; ANA: antinuclear antibodies; anti-ENA: anti-extractable nuclear antigen antibodies; AMA: antimitochondrial antibodies; ASMA: anti-smooth muscle antibodies; anti-LKM1: anti-liver/kidney microsome type 1 antibodies; ALT: alanine aminotransferase; AP: alkaline phosphatase; US: ultrasonography; CT: computed tomography; EMG: electromyography; RA: rheumatoid arthritis; SS: Sjögren's syndrome

## Differential diagnosis

The term 'essential' MC was originally referred to autonomous disease when other well known systemic, infectious or neoplastic disorders have been ruled out by means of a wide clinico-serological work-up [5]. However, in some patients a definite diagnosis may be difficult because of the clinical polymorphism of the disease. Moreover, the association of MC with HCV infection may further complicate the differential diagnosis with other immunological HCV-related disorders: there is a frequent clinico-pathological overlapping among different HCV-related disorders. Cryoglobulinemic syndrome can represent a crossroads between some autoimmune diseases (autoimmune hepatitis, Sjögren's syndrome, polyarthritis, glomerulonephritis, thyroiditis, type 2 diabetes, *etc*.) and malignancies (B-cell lymphomas, hepatocellular carcinoma) [3,11,12,25,28,87-98]. It is possible to observe in the same patient a slow progression from mild HCV-associated hepatitis to various extrahepatic manifestations (arthralgias, sicca syndrome, Raynaud's phenomenon, RF positivity, *etc*.), and ultimately to overt MC syndrome with typical clinico-serological manifestations. In only a minority of MC patients a malignancy may develop, generally after a long lasting follow-up period [3,11,12,24,25]. Therefore, a careful patient evaluation is necessary for a correct diagnosis of MC syndrome, particularly with regards to other RF-positive, systemic rheumatic disorders such as rheumatoid arthritis (RA) and primary Sjögren's syndrome (pSS) (Fig. 7).

While arthralgias are one of the most frequent symptoms, clear signs of sinovitis are quite rare. Generally, patients develop mild, non-erosive oligoarthritis [28,90], often sensitive to low doses of corticosteroids with or without hydroxychloroquine. In contrast, a rheumatoid-like polyarthritis is more frequent in patients with HCV-related hepatitis without MC syndrome [28]. In patients with HCV-associated MC and symmetrical, erosive polyarthritis the diagnosis of overlapping MC/RA syndrome can be suspected. In these cases, the detection of serum anti- cyclic citrullinated peptide antibodies, markers of classical RA, may represent a useful diagnostic tool [91,92].

Almost half MC patients complain of sicca syndrome; however, only a few cases meet the current criteria for the classification of pSS. MC and pSS may share various symptoms: xerostomia and/or xerophthalmia, arthralgias, purpura, RF and serum cryoglobulins, and the possible complication with B-cell lymphoma [3,11,12,25,87,93-98]. However, a careful patient clinical assessment is usually sufficient for a correct diagnosis in the large majority of cases by considering some important findings: histopathological alterations of salivary glands and specific autoantibody pattern (anti-RoSSA/LaSSB) of pSS are rarely found in MC patients; conversely, HCV infection, cutaneous leukocytoclastic vasculitis, and visceral organ involvement (renal, liver) are seldom recorded in primary Sjögren's syndrome (Fig. 7). Given the above considerations, it has been recently proposed that the presence of HCV infection *per se *should be considered exclusion criteria for the classification of pSS [99,100]. In rare cases in which the differential diagnosis may result very difficult, particularly in HCV-negative individuals, it might be correct to classify the disorder as overlap syndrome. However, patients with MC/pSS overlap syndrome are often characterized by more severe clinico-prognostic evolution; they show a significant low rate of anti-RoSSA/LaSSB along with a high prevalence of mixed cryoglobulinemia, hypocomplementemia, systemic autoimmune manifestations, and complicating lymphomas (94, 97, 98). In the clinical practice, this particular condition could be better regarded as vasculitic syndrome with relevant implications on the patient monitoring and treatment [11,24,25].

Finally, autoimmune hepatitis, the old 'lupoid' hepatitis, may be associated to HCV infection, mainly in the same countries where HCV-associated MC is more frequently found [88]. Moreover, it may share with MC syndrome a number of extrahepatic symptoms, including serum mixed cryoglobulins [89]. The differential diagnosis between these two conditions may be problematic: some typical features of MC (leukocytoclastic vasculitis, hypocomplementemia, glomerulonephritis), as well as the presence of serum autoantibodies commonly found in autoimmune hepatitis (anti-smooth muscle antibodies) can be taken into account.

## Management

**Figure 8:**
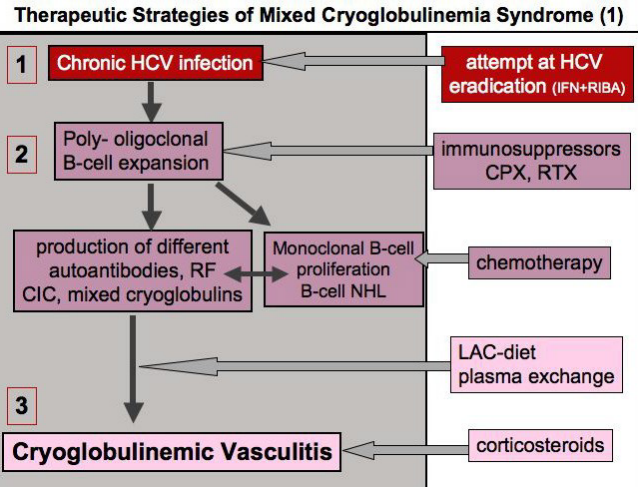
**Treatment of mixed cryoglobulinemia (MC) syndrome according to the etiopathogenesis of the disease (1)**. MC is the result of a multistep process including three main clinico-pathological levels: 1) chronic HCV infection, 2) B-lymphocyte proliferation, and 3) immune-complex-mediated vasculitis (cryoglobulinemic vasculitis). Following the cascade of events leading from HCV infection to overt vasculitic syndrome, we can treat the patients at different levels by means of etiologic, pathogenetic, and/or symptomatic therapies (see also Fig. 6, 9, and 10). RF: rheumatoid factor; CIC: circulating immune-complexes; LAC-diet: low-antigen-content diet. (modified from [25]: Ferri C and Mascia MT, *Curr Opin Rheumatol *2006, 18: 54–63, with permission from Lippincott Williams & Wilkins).

**Figure 9:**
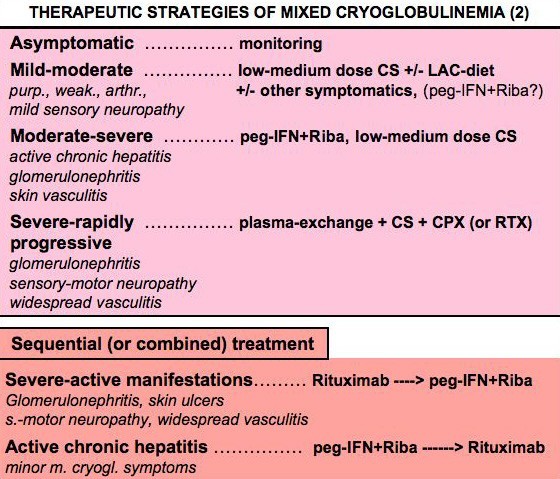
**Therapeutic strategy according to activity/severity of mixed cryoglobulinemia (MC) syndrome (2)**. Therapeutic strategy of mixed cryoglobulinemia (MC) syndrome (or cryoglobulinemic vasculitis) should be decided on the basis of the activity/severity of clinical symptoms and tailored for the single patient: in asymptomatic patients a careful monitoring is often sufficient; in those with moderate-severe manifestations, mainly in the presence of active chronic hepatitis, an attempt to eradicate the HCV infection should be carried out; particularly severe, rapidly progressive complications must be treated with more aggressive treatments, as in other systemic vasculitides. Sequential treatment schedules can be employed in individuals with particularly aggressive manifestations and/or partial response (clinical, pathological, or virological) to traditional treatments (see also Fig. 8 and 10). Purp.: purpura; weak.: weakness; arthr.: arthralgias; CPX: cyclophosphamide; CS: corticosteroids; LAC-diet, low-antigen-content diet; MPGN, membranoproliferative glomerulonephritis; peg-IFN: pegylated interferon-alpha; RIBA: ribavirin.

**Figure 10:**
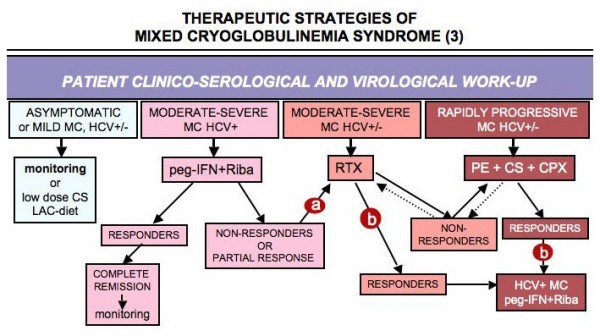
**Flow chart of therapeutic strategies according to activity/severity of mixed cryoglobulinemia (MC) syndrome (3)**. HCV-positive MC patients with moderate-severe manifestations, mainly in the presence of active chronic hepatitis, antiviral treatment with pegylated interferon-alpha (peg-IFN) + ribavirin (RIBA) can be tried after exclusion of possible contraindications. In non-responders or in those with partial response (virological), a treatment with rituximab (RTX) can be proposed (a). Conversely, in HCV+ MC patients usefully treated with rituximab or other anti- inflammatory/immunosuppressive therapies, an attempt to eradicate HCV infection can be done (b). Finally, combined high dose corticosteroids (CS), plasma exchange (PE), and cyclophosphamide (CPX) are the first line treatment in life-threatening, rapidly progressive complications in both HCV+ and HCV- cryoglobulinemic vasculitis (see also Fig. 8 and 9). LAC-diet: low-antigen-content diet.

Considering its complex etiopathogenesis and clinical polymorphism, the treatment of MC syndrome is particularly challenging. For a correct therapeutic approach, three important factors (Fig. 6), namely, the HCV infection, the presence of autoimmune disorder, and the possible neoplastic complications should be considered [3,25]. Following the main steps of the etiopathogenetic process, from HCV infection to B-lymphocyte proliferation, and lastly to cryoglobulinemic vasculitis (Fig. 8), the disease can be treated at different levels by means of etiologic, pathogenetic, and/or symptomatic therapies. Since HCV represents the main causative factor of the disease by exerting a chronic stimulus on the immune-system [3,8-12,14,24], an attempt at HCV eradication by alpha-interferon treatment should be done in all cases of HCV-associated MC [3,11,12,24,105-109]. However, the beneficial effect observed with this drug is often transient and possibly associated with important immune-mediated side effects such as peripheral sensory-motor neuropathy, thyroiditis, and rheumatoid-like polyarthritis [51-54]. Probably, in predisposed subjects alpha-interferon (which is both antiviral and immunomodulating agent) can trigger or exacerbate some pre-existing, often subclinical, symptoms. Unfortunately, there are no available parameters for predicting this harmful complication; thus, alpha-interferon therapy should be avoided at least in those patients with clinically evident peripheral neuropathy. On the whole, the usefulness of alpha-interferon treatment in MC patients is limited by the low rate of responders and frequent side effects. The association of alpha-interferon and ribavirin might achieve the eradication of HCV infection in a higher number of treated subjects, even if the long-term effects are inconstant and often unpredictable [3,11,12,24,105-114]. After careful patient's evaluation of contraindications and/or possible side effects, a standard treatment with alpha-interferon and ribavirin [111] should be attempted in MC patients with prevalent clinico-pathological findings of chronic hepatitis (Fig. 9, 10). Controlled clinical trials might establish the actual usefulness of antiviral therapy in HCV-associated MC and better evaluate the predictive markers of response to treatment (HCV genotypes, host HLA alleles?). Hopefully, with the rapid growth of molecular biology a vaccine against HCV might be available in the near future. A vaccine-based therapy [115] with recombinant HCV proteins in HCV-infected individuals could be able to prevent the progression of viral infection and possibly to interrupt the self-perpetuating autoimmune mechanism underlying the MC.

In patients with 'essential' MC the immunosuppressive treatment, *i.e*. cyclophosphamide or rituximab, is still the first-line intervention (Fig. 9, 10). For HCV-associated MC, immunomodulating/immunosuppressive treatments should be considered, especially in patients with more severe complications. These treatments include steroids, low-antigen-content (LAC) diet, plasma exchange, and immunosuppressors [3,11,12,24,116-122]. In particular, both traditional plasma exchange and double-filtration plasma exchange are able to markedly reduce the levels of circulating immune-complex, especially the cryoglobulins [24,121,122]. Oral cyclophosphamide (50–100 mg/day for 2–6 weeks) during the tapering of apheretic sessions can reinforce the beneficial effect of plasma exchange; moreover, it can prevent the rebound phenomenon that may be observed after the aphaeresis discontinuation [24]. Plasma exchange is particularly useful in severe MC complications such as active membranoproliferative glomerulonephritis (Fig. 8, 9, 10).

LAC-diet is a particular dietetic treatment that can improve the clearance circulating immune-complexes by restoring the activity of the reticulo-endothelial system, overloaded by large amounts of circulating cryoglobulins [122,123]. LAC-diet has been designed to reduce the input of alimentary macromolecules crossing the mucosal barrier of the gut; some foods, particularly dairy products and eggs, present a potential antigenic activity, and consequently might be involved in the pathogenesis of some diseases in humans. The reduction of the alimentary input of macromolecules directed to mononuclear phagocytic system may improve its function in those conditions characterized by abnormal endogenous production of immune-complexes responsible for organ damage, *i.e*. mixed cryoglobulinemia and other immune-complex mediated diseases. An impaired function of mononuclear phagocytic system has been also demonstrated in patients with IgA nephropathy [123]. Given the above pathogenetic considerations, LAC-diet has been usefully employed in patients with clinically mild MC symptoms and IgA nephropathy (reduction of 24-hour proteinuria). Usually, LAC-diet and/or low dosage of steroids (6-methyl-prednisolone 2–4 mg/day) may be sufficient to improve mild manifestations (arthralgias, sporadic purpura, *etc*.) of the MC (Fig. 8, 9, 10); patients with mild-moderate symptoms, such as palpable purpura, are particularly sensitive to the smallest variations of daily steroid dosage (1–2 mg).

In clinical practice, MC treatment should be tailored for the single patient according to the severity of clinical symptoms (Fig. 9, 10). Therefore, patients with severe vasculitic manifestations must be promptly treated with high doses of steroids and/or plasma exchange and/or cyclophosphamide or rituximab. Some recent clinical studies suggested that sequential or combined antiviral/immunosuppressive treatment could represent a rather useful therapeutic strategy [124,125]. The rationale of such aggressive therapies could be particularly indicated in MC patients with major clinical manifestations and partial/transitory remission after standard treatments [25,124,125]. On the contrary, clinically asymptomatic patients usually do not need any treatment, even in the presence of high levels of cryocrit. In all cases, a careful clinical monitoring of the disease is mandatory, with particular attention to neoplastic complications. Preliminary studies suggested the synergic affect of some therapies in association with antiviral treatment, namely the cyclosporine A or tumor necrosis factor inhibitors, in the setting of chronic hepatitis type C [126-128]. These agents may contribute to viral clearance and/or to improve the immune-mediated hepatic and extrahepatic inflammatory manifestations; future clinical trials might evaluate their potential usefulness even in MC, possibly in selected patient subsets.

## Prognosis

The natural history of MC is not predictable and strongly depends on concomitant diseases and complications and response to treatment. Morbidity due specifically to cryoglobulinemia may also be significant (infections, cardiovascular diseases, progressive renal failure, advanced neuropathy). The overall prognosis is worse in patients with renal disease, liver failure, lymphoproliferative disease, and malignancies. Mean survival is estimated to be approximately 50–60% at 10 years after diagnosis [24]. Careful monitoring of life-threatening MC complications (mainly nephropathy, widespread vasculitis, and B-cell lymphoma or other malignancies) should be carried out in all MC patients.

## Unresolved questions

MC was first described as distinct disorder in 1966 [5]; 25 years later the discovery of the strong association between MC and HCV infection [3,11,12,25,70] represented a decisive contribute for a better understanding of the etiopathogenetic mechanisms responsible for the disease, and consequently for an adequate therapeutic strategy.

However, the following points remain still to be clarified:

1- Since the etiopathogenesis of HCV-associated MC syndrome is a multifactorial and multistep process, two important aspects need further investigations: a) HCV may represent the simple triggering factor or it could also contribute to self-perpetuating mechanism of the disease: b) nature and role of other etiopathogenetic co-factors;

2- The etiopathogenesis of 'essential' MC syndrome;

3- The actual role of HCV eradication on the natural history of HCV-associated MC syndrome;

4- The usefulness of sequential or combined antiviral/immunesuppressive treatments compared to traditional therapeutic approach.

## Abbreviations

Ig: immunoglobulin; RF: rheumatoid factor; MC: mixed cryoglobulinemia; HCV: hepatitis C virus; MPGN: membranoproliferative glomerulonephritis; B-CLL: B-cell chronic lymphocytic leukemia; Ic: immunocytoma; MLDUS: monotypic lymphoproliferative disorder of undetermined significance; HBV: hepatitis B virus; PCR: polymerase chain reaction; VLDL: very low-density lipoprotein; MALT: mucosa-associated lymphoid tissue; HIV: human immunodeficiency virus; RA: rheumatoid arthritis; pSS: primary Sjögren's syndrome; LAC-diet: low-antigen-content diet.

## Competing interests

The author declares that they have no competing interests.
